# Three-Dimensional Evaluation of Bifid Condyle by Cone-Beam Computed Tomography

**DOI:** 10.7759/cureus.46529

**Published:** 2023-10-05

**Authors:** Ezhilarasi Arumugam Venkatachalam Sargurunathan, Ignatious Jeba Mary Rajkumar, Ramachandra Venkatesha Reddy, Saramma Mathew Fenn, Karthik Rajaram Mohan

**Affiliations:** 1 Oral Medicine and Radiology, Vinayaka Mission's Sankarachariyar Dental College, Vinayaka Mission's Research Foundation (Deemed to be University), Salem, IND

**Keywords:** temporomandibular joint, temporomandibular pain, developmental anomalies, cone-beam computed tomography (cbct), bifid mandibular condyle

## Abstract

Bifid condyle is a rare developmental anomaly that results from an obstructed blood supply during its development. Bifid condyle is more often unilateral, although bilateral. A case of a bifid condyle is evaluated three-dimensionally in three orthogonal planes namely coronal, sagittal and axial sections. The etiology, clinical features, diagnostic, non-surgical and surgical treatment modalities of bifid condyle are discussed.

## Introduction

A bifid condyle is a rare developmental anomaly affecting the temporomandibular joint's condyle [1]. A bifid condyle has a vertical notch, depression or cleft in the center of the condylar head, seen in the coronal plane, resulting in the appearance of a "double condylar head" [1]. This condition is rare and most often unilateral, although it occurs bilaterally [1]. It is usually seen as an incidental finding during routine conventional orthopantomography, anteroposterior skull projections and cone-beam computed tomography scans [1]. Some patients have clinical signs of dysfunction of the temporomandibular joint, such as pain and clicking noise [1]. The bifid condyle usually has a depression or notch in the superior condylar surface resulting in a heart-shaped condyle [1]. The depression or notch is usually variable from complete duplication of the condylar head in the sagittal plane or as a minor groove that traverses only a short distance mimicking a vertical fracture [1]. The orientation of the condyle may be mediolateral or anteroposterior [1]. In addition, the mandibular or articular fossa may remodel to accommodate altered condylar morphology [1].

## Case presentation

**Figure 1:**
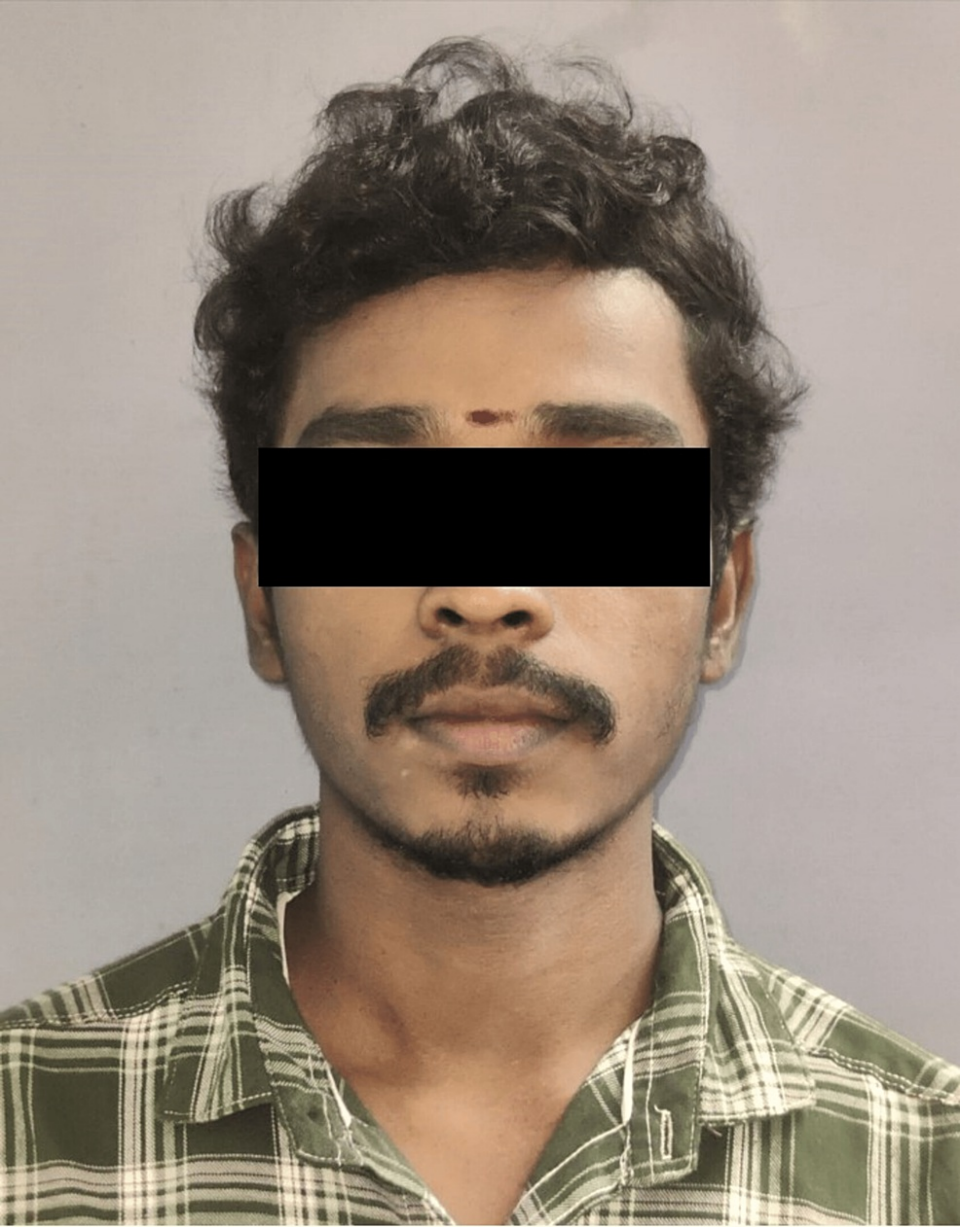
Extraoral clinical photograph did not reveal any facial asymmetry

A 26-year-old male reported to the dental outpatient department with a chief complaint of pain in his left temporomandibular joint region for the past two days. History revealed the patient had clicking of the temporomandibular joint for the past six months during movements of the mandible, and there was no history of trauma to the temporomandibular joint. Further probing of his family history revealed he was born between parents of a non-consanguineous marriage. His medical history was non-contributory. On probing, his past surgical history among his family members revealed he was born of non-forceps delivery. In addition, extraoral examination revealed the absence of facial asymmetry due to any swelling or any visible scars near the temporomandibular joint (Figure 1).

On further examination, the mandible deviates to the left side during the initial mouth opening and returns to the centric relation position during maximum mouth opening (Video 1).

**Video 1:** Deviation of the mandible to the left side during mouth opening

**Figure 2:**
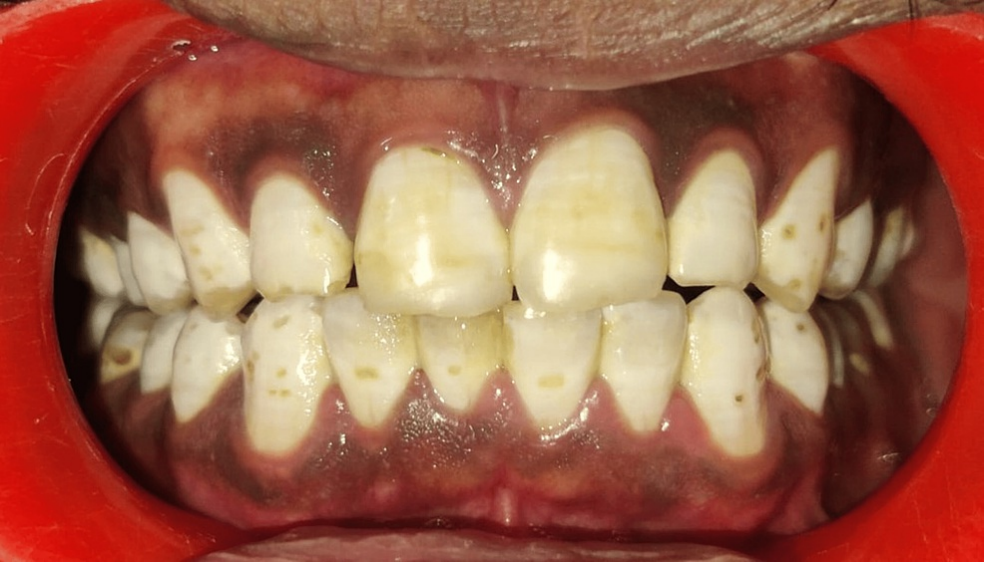
Intraoral examination revealed no occlusal derangements and generalized intrinsic chalky white discoloration with discrete mottling or pitting in all teeth

On palpation, no tenderness present on temporomandibular joint regions. The intraoral examination did not reveal any occlusal derangements. However, intrinsic chalky white enamel areas and discrete enamel mottling are present on all teeth (Figure 2).

**Figure 3:**
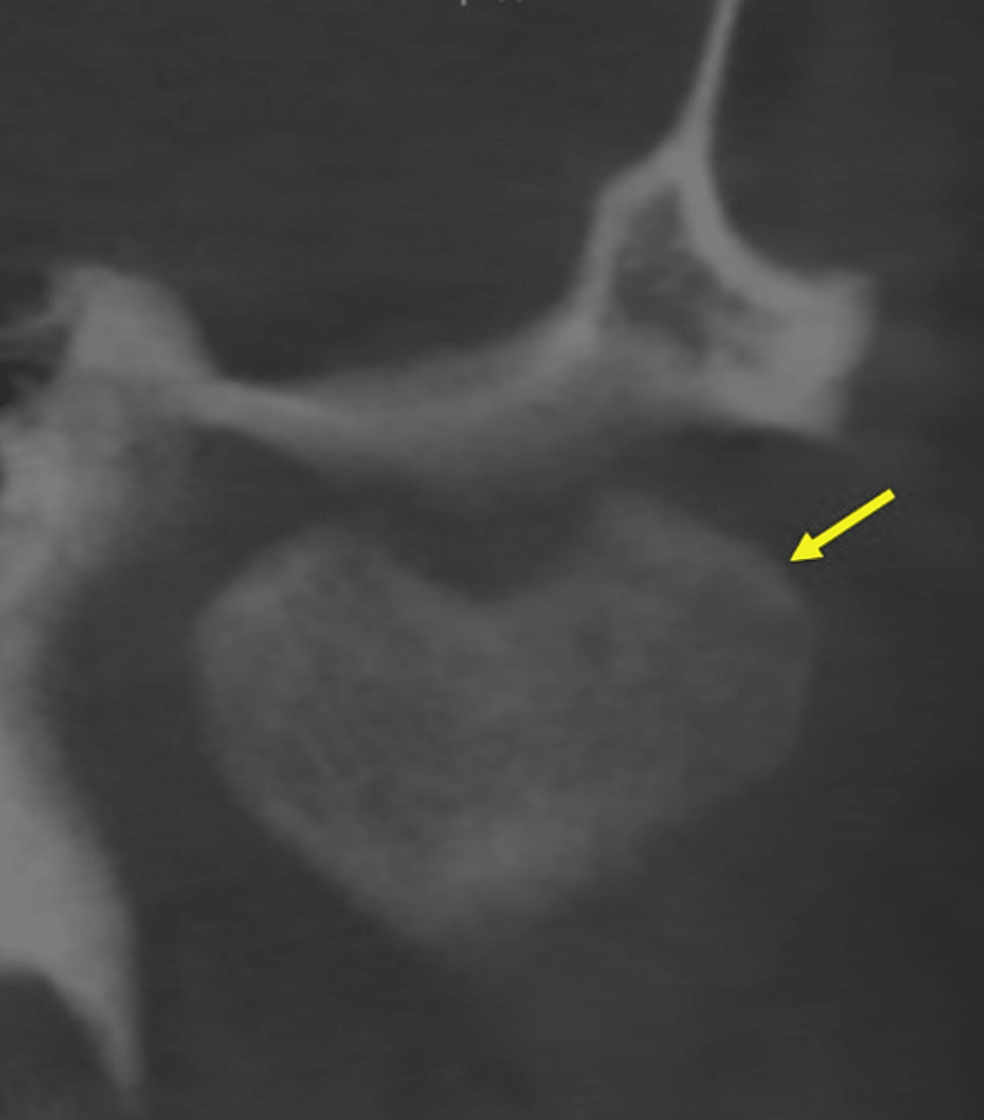
Cone-beam computed tomography (CBCT) coronal section of the left temporomandibular joint (TMJ) revealed the bifid condyle resembling a heart shape (yellow arrow)

The provisional diagnosis was made as internal derangement of the articular disc of the left temporomandibular joint (TMJ) region and dental fluorosis. The anterior disc displacement with reduction type of internal derangement is also associated with a second reciprocal click, which is not appreciable in clicking of TMJ caused by bifid mandibular condyle. Magnetic resonance imaging (MRI) of TMJ with 3 Tesla helps to study the finer details and changes in the articular disc in such instances. Sagittal section cone-beam computed tomography (CBCT) revealed two condylar heads on the left temporomandibular joint (Figure 3).

**Figure 4:**
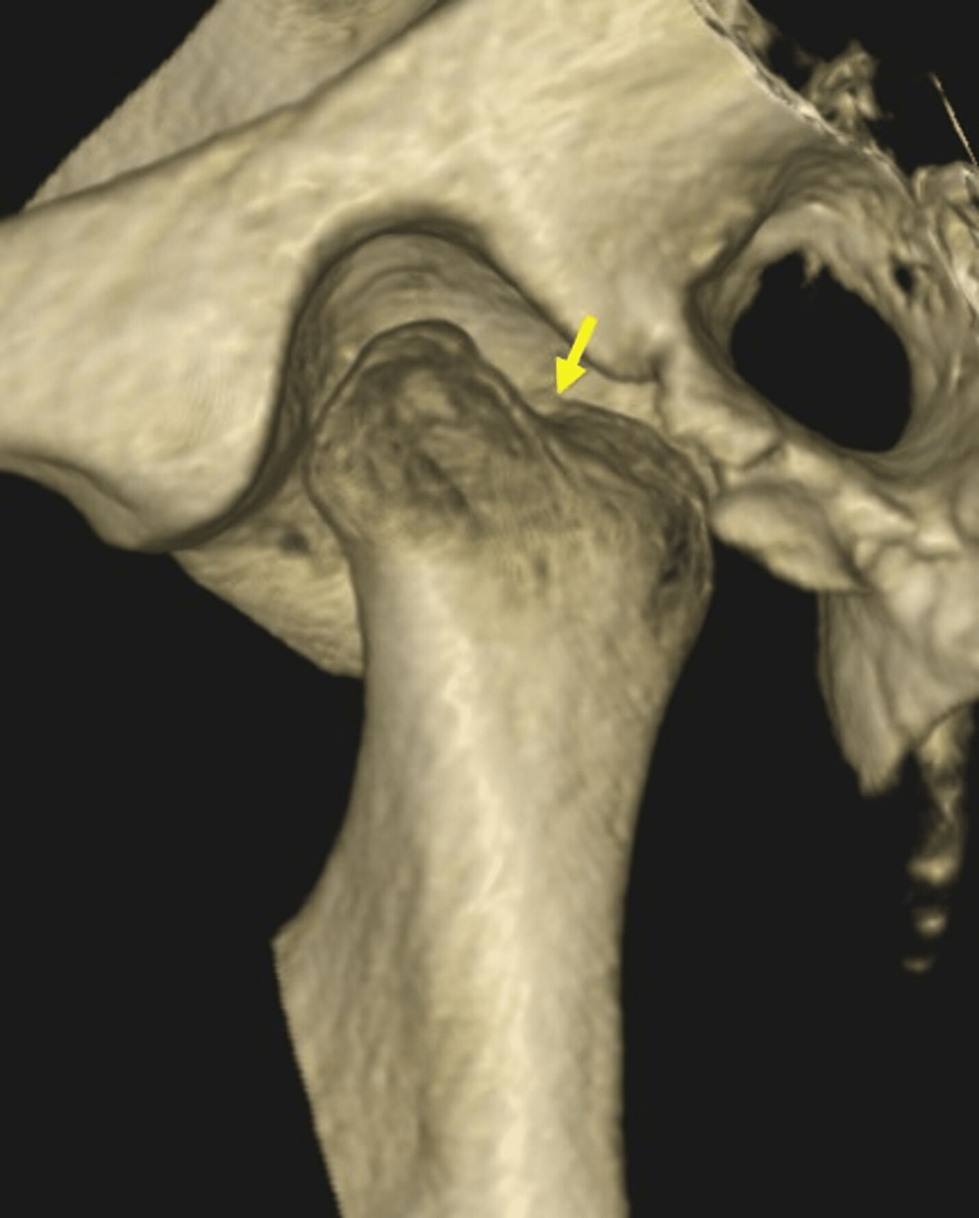
Three-dimensional reconstructed cone-beam computed tomography (CBCT) revealed the bifid condyle involving the left temporomandibular joint (TMJ) (yellow arrow)

A three-dimensional reconstructed cone-beam computed tomography (CBCT) image revealed a bifid condyle involving the left temporomandibular joint (TMJ) (Figure 4).

A final diagnosis of the bifid condyle was made. The patient was counseled for surgical procedures but was afraid to undergo surgery, so he refused surgical treatment. Instead, the patient has been prescribed a tablet of ketorolac tromethamine 10 mg twice daily for three days to provide symptomatic pain relief. In a follow-up after a week, the patient was relieved from his pain over the left temporomandibular joint (TMJ).

## Discussion

Definition

Bifid condyle is a rare developmental anomaly that affects the condylar head of the temporomandibular joint (TMJ), resulting in bifid (two) condylar heads resembling heart-shaped condyle [1].

Synonyms

The bifid condyle is also known as the double-headed condyle.

Etiology

The etiology of the bifid condyle is unknown and controversial. The embryonic theory states that bifid condyles occur when the blood supply to the condyle is blocked or when fibrous septa persist. Some authors attribute the formation of bifid condyle due to underlying trauma [2]. Trauma, such as birth trauma, condylar fractures or surgical condylectomy, may also cause joint integrity disruption or dislocation. Minor trauma to the condylar growth center results in a bifid condyle [2]. The lateral pterygoid muscle plays a crucial role in creating a BMC by determining the direction of the fractured condylar piece [3]. Due to lateral pterygoid muscle activity, the condylar neck fracture leads to an anteromedial displacement. By the action of the lateral pterygoid muscle, the condyle is displaced anteromedially in a fracture of the condylar neck, and a new condylar head forms at the normal anatomical site through metaplasia of local fibroblasts, while the displaced head undergoes resorption. A condylar fracture may lead to the development of anteroposterior bifid condylar heads. When a sagittal fracture through the condylar head occurs, a sagittal condylar dividing will produce medial and lateral heads. Sagittal fractures can also result in anteromedial displacement of the medial head by the lateral pterygoid muscle, but the displacement is less than condylar fractures. Bifid condyles can develop after condyle trauma when remodeling capacity is insufficient, and it appears that severity, site and location of trauma, as well as the position of the lateral pterygoid muscles, all play an important role in determining the appearance and orientation of the condyle. A new condylar head appears by metaplasia in a correct anatomic position, while the displaced condyle begins to resorb [3]. As a result, only the posterior condyle is functional out of the two [3]. Remodeling the condylar fragment head can lead to a bifid condyle [3]. Some believed that obstructed blood supply during its development led to the bifid condyle [3]. The condylar cartilage has a fibrous septa partition during its development [3]. The persistence or loss of blood supply in the septa during the development of the condyle leads to a bifid condyle [3]. Some believe it is an embryopathy, where a combination of teratogenic agents and misdirection of muscle fibers leads to abnormal bone formation [3]. Exposure to teratogenic agents such as hydroxamic acid and N-Methyl-N-nitrosourea has been implicated in forming bifid condyle in animals such as rats at various stages of pregnancy [3]. Infection from arthritis can also lead to bifid condyle [3].

Epidemiology

The prevalence of bifid condyle is 0.31% to 1.82%. No age predilection exists. However, bifid condyle occurs in age groups of six to 35 years [4]. The study by Nikolova et al. reported an incidence of unilateral bifid condyle observed in four (0.8%) of 500 dry mandibles, with two (0.4%) occurring in the right condyle and two (0.4%) occurring in the left condyle [4]. The prevalence rate of bifid condyles, as studied from orthopantomography and cone-beam computed tomography of paranasal sinuses, was 3.5% and 4.53%, respectively [5,6] (Table 1).

**Table 1:** The prevalence rate of bifid condyles CBCT: cone-beam computed tomography.

Author	Year	Location	Radiographic examination used	Prevalence (%)
Khojastepour et al. [5]	2015	Shiraz, Iran	CBCT-paranasal sinus view	The study enrolled 309 patients with condyles visible on CBCT scans: 170 (55%) were females and 139 (45%) were males with a mean age of 39.43±9.7 years. Fourteen cases (4.53%) had bifid mandibular condyles.
Haghnegahdar et al. [6]	2014	Shiraz, South Iran	Orthopantomography	A panoramic view of 1,000 individuals (767 females and 233 males) above 18 years of age showed that 35 (3.5%) have bifid condyle.

Classification

Bifid condyle is also known as double-headed condyles. The classification of the bifid condyle is enumerated in Table 2 [7].

**Table 2:** Classification of bifid condyle

Guven O classification of Bifid condyle	
Type I	No history of trauma (non-traumatic), asymptomatic/non-symptomatic mediolateral bifid mandibular condyle
Type II	History of trauma (traumatic), mediolateral/anteroposterior bifid mandibular condyle
Group a	a) With a Y-shaped condyle secondary to an intracapsular or vertical condyle trauma
Group b	b) With two separate and anteroposteriorly located condyles, where the formation of the anteroposterior condyle might be due to insufficient remodeling of the ramus/condyle of the displaced or dislocated subcondylar fracture resulting from the force exerted by the lateral pterygoid muscle

Clinical features

The clinical features of bifid condyle vary from asymptomatic cases to progressive pain over the temporomandibular joint (TMJ), facial asymmetry, stuck disc, progressive deterioration with a reduction in mouth opening and lateral mandibular excursions [7]. A bifid condyle was first described in 1941 by Hrdlicka. Hrdlicka identified 21 bifid mandibular condyles (BMCs) (18 unilateral and three bilateral) in dry skulls at the Smithsonian Institution in Washington, DC, among a number of unspecified dry skulls [7]. Schier reported the first case of the bifid condyle in 1948 [7]. The various research studies on case reports of bifid condyle are given in Table 3.

**Table 3:** Literature reviews on case studies on bifid condyle CBCT: cone-beam computed tomography, TMJ: temporomandibular joint, BMD: bifid mandibular condyle.

Author	Year	Age	Clinical description
Çelik et al. [8]	2022	6-year-old child	Bilateral bifid mandibular condyle associated with ankylosis reduces the quality of life.
Michalski et al. [9]	2022	9-year-old boy	Despite the fact that BMC is rare and poorly understood, it should be taken into account when there is a TMJ pathology.
Bettoni et al. [10]	2021	20-year-old patient	Bifid condyle is a relatively rare condition that is becoming more prevalent, but clinicians should be aware of its real pathophysiology so they can exclude a possible misdiagnosis.
Periasamy and Kumar [11]	2021	38-year-old male	The condition of the bifid mandibular condyle is extremely rare.
Coclici et al. [12]	2020	29-year-old male	In diagnosing bifid mandibular condyle, CT/CBCT provides the most accurate assessment of morphological aspects like condyle shape, size and orientation of condylar angle, joint position and glenoid fossa depth.
Miranda et al. [13]	2019	17-year-old male	Pain and mastication difficulty due to a severe limitation in mouth opening.
Desai [14]	2019	18-year-old female	Atypical oculo-auriculo-vertebral spectrum (OAVS) with radial defects, unilateral bifid condyle and taurodontism.
Woo et al. [15]	2016	9-year-old female	Bifid mandibular condyle can occur as a result of fracture of condylar head.
Neelakandan and Bhargava [16]	2013	14-year-old male	Bifid condyle associated with condylar hyperplasia.

Diagnosis

Bifid condyles are better appreciated more commonly by specialized radiographic techniques like computed tomography, magnetic resonance imaging and cone-beam computed tomography imaging techniques than conventional orthopantomography. Computed tomography and cone-beam computed tomography provide three-dimensional visualization of condyles in three orthogonal planes namely the coronal, sagittal and axial sections. In conventional radiographs like panoramic radiography, bifid condyle may not be apparent sometimes, since orthopantomography is a two-dimensional imaging technique, where the presence of an aberrant extra condyle is masked by superimposition or overlapping of the functional posteriorly placed condyle over the anterior head [8].

Treatment

The treatment modalities for bifid condyle depend on the clinical signs and symptoms [8]. No treatment is indicated in asymptomatic cases of the bifid condyle [8]. However, if clicking of the temporomandibular joint (TMJ) persists in cases of the bifid condyle, condylectomy or eminectomy is suggested. The various non-surgical and surgical management for bifid condyle is discussed in Table 4.

**Table 4:** Non-surgical and surgical management of bifid condyle

Non-surgical and surgical management of bifid condyle	
Non-surgical management	For bifid condyle without temporomandibular joint ankylosis
Non-steroidal anti-inflammatory drugs	Tablet.Piroxicam 20 mg once daily or Tablet.Ketorolac 10 mg twice daily for palliative pain relief in mild symptomatic cases of pain in patients with bifid condyle
Splints: anterior repositioning splints	Splints help to reposition the jaw
Physiotherapy	Increase the laxity of lateral pterygoid muscles attached to the articular disc
Surgical management	Indicated only for post-traumatic cases of bifid condyle associated with temporomandibular joint ankylosis
Condylectomy	Surgical resection of the abnormally positioned additional condyle
Gap arthroplasty	A surgical procedure of removal of the ankylosed part of the condylar head to create a gap of 10-15 mm between the articular (glenoid) fossa and ramus of the mandible without the use of any interpositional material in cases of ankylosis associated with bifid condyle secondary to trauma
Interpositional arthroplasty	Creates a gap of less than 5 mm. Helps to restore mandibular deviation in the bifid condyle
Coronoidectomy	Surgical procedure that involves removal of coronoid process of the mandible in patients with restricted mouth opening due to bifid condyle
Eminectomy	Surgical procedure that reduces articular eminence. Indicated to relieve stuck disc caused by increased articular eminence in the bifid condyle

The uniqueness of the case report is that it describes a rare TMJ developmental anomaly, which usually goes unnoticed and is mostly discovered incidentally by routine radiographic evaluation. The case describes a bifid condyle in the absence of any occlusal derangement. This case report enumerates light on the etiology, clinical features, diagnostic and treatment modalities of bifid condyle that every dentist must know. A non-surgical pharmacotherapeutic treatment modality was provided for this rarest developmental anomaly affecting the condyle in our case. Hence, invasive surgical treatment, which can also be prone to infection, is prevented.

## Conclusions

Bifid condyle is a rarest developmental variation resulting in a bifid condylar head resembling a heart-shaped condylar head. Bifid condyle is diagnosed chiefly by incidental radiological evaluation by conventional radiographs like orthopantomography, trans-pharyngeal projection of temporomandibular joint and specialized radiographic techniques like computed tomography (CT), magnetic resonance imaging and cone-beam computed tomography (CBCT). A careful radiological evaluation by cone-beam computed tomography helps diagnose cases of bifid condyle. Bifid condyle, if left untreated, can cause pain or clicking of TMJ, reduced mouth opening and permanent luxation. Therefore, dentists must be aware of the clinical features of the bifid condyle to diagnose this rare anomaly and also provide earlier treatment.
